# ϒ-secretase and LARG mediate distinct RGMa activities to control appropriate layer targeting within the optic tectum

**DOI:** 10.1038/cdd.2015.111

**Published:** 2015-08-21

**Authors:** P Banerjee, H Harada, N G Tassew, J Charish, D Goldschneider, V A Wallace, S Sugita, P Mehlen, P P Monnier

**Affiliations:** 1 Genetics and Development and Vision Divisions, Toronto Western Research Institute, Krembil Discovery Tower, KDT-8-418, 60 Leonard Street, Toronto, ON, Canada M5T 2S8; 2 Department of Physiology, Faculty of Medicine, University of Toronto, 1 King's College circle, Toronto, ON, Canada M5S 1A8; 3 Apoptosis, Cancer and Development Laboratory- Equipe labellisée ‘La Ligue', Centre de Cancérologie de Lyon, INSERM U1052-CNRS UMR5286, Université de Lyon, Centre Léon Bérard, Lyon 69008, France; 4 Department of Ophthalmology and Vision Science, Faculty of Medicine, University of Toronto, 340 College Street, Toronto, ON, Canada M5T 3A9

## Abstract

While a great deal of progress has been made in understanding the molecular mechanisms that regulate retino-tectal mapping, the determinants that target retinal projections to specific layers of the optic tectum remain elusive. Here we show that two independent RGMa-peptides, C- and N-RGMa, activate two distinct intracellular pathways to regulate axonal growth. C-RGMa utilizes a Leukemia-associated RhoGEF (LARG)/Rho/Rock pathway to inhibit axonal growth. N-RGMa on the other hand relies on ϒ-secretase cleavage of the intracellular portion of Neogenin to generate an intracellular domain (NeICD) that uses LIM-only protein 4 (LMO4) to block growth. In the developing tectum (E18), overexpression of C-RGMa and dominant-negative LARG (LARG-PDZ) induced overshoots in the superficial tectal layer but not in deeper tectal layers. In younger embryos (E12), C-RGMa and LARG-PDZ prevented ectopic projections toward deeper tectal layers, indicating that C-RGMa may act as a barrier to descending axons. In contrast both N-RGMa and NeICD overexpression resulted in aberrant axonal-paths, all of which suggests that it is a repulsive guidance molecule. Thus, two RGMa fragments activate distinct pathways resulting in different axonal responses. These data reveal how retinal projections are targeted to the appropriate layer in their target tissue.

Over the last 20 years, a great deal of progress has been made in understanding how retinal axons establish anterio-posterior as well as dorso-ventral retino-topic maps.^1, 2, 3, 4^ Multiple protein gradients in the optic tectum (OT) provide positional information that allow for precise targeting of retinal axons. When they reach the tectum, retinal axons will first extend within the most superficial layer of the OT, the stratum opticum (SO). Once they reach the appropriate tectal-coordinate, retinal axons turn into deeper layers to establish terminal arbors with the stratum griseum et fibrosum superficiale (SGFS) layer. Very little is known on (i) how axons make the decision to turn into deeper tectal layers and (ii) the mechanisms that restrict terminal arbors from overshooting their laminar destination. During the formation of anterio-posterior projections, fibers may overshoot their target layer, subsequent elimination of these overshoots will ensure that they never pass the g layer of the SGFS.^5^

The Repulsive Guidance Molecule a (RGMa) is expressed in a high-posterior low-anterior gradient in the OT, and possesses activities similar to some members of the Ephrin family, inhibiting outgrowth of temporal retinal ganglion cell (RGC) axons.^2, 4, 6^ Axonal growth inhibition occurs upon binding of RGMa to its transmembrane receptor Neogenin, which is expressed in a high-temporal low-nasal gradient in RGC axons.^7^ In the chick, gain- and loss-of-function analyses have shown that perturbing the RGMa gradient in the OT causes pathfinding mistakes for temporal axons.^8, 9^ The majority of the studies performed with RGMa were performed with engineered full-length protein. However, recent studies have demonstrated that RGMa processing by proteases generates multiple RGMa peptides that govern axonal growth. Despite no sequence homology, N- and C-RGMa fragments bound the same Fibronectin domain in Neogenin and blocked outgrowth.^10, 11, 12^

While guidance molecules control topographic mapping, it is not known whether or not they influence turning into deeper tectal layer. We were puzzled by the fact that independent N- and C-RGMa peptides compete for binding to the same receptor to lead to the same axonal blockage. To address the possibility that these peptides may have distinct activities, we performed a closer investigation. We provide evidence that C- and N-RGMa use two distinct pathways to regulate different aspects of retino-tectal projections. C-RGMa inhibits axonal-outgrowth but does not influence axonal guidance, whereas N-RGMa does both. C-RGMa prevents projections into deeper tectal layers, whereas N-RGMa promotes deeper projections. Furthermore, we show that C-RGMa activates the Leukemia-associated RhoGEF (LARG)/Rho/Rock pathway to block axonal growth, whereas N-RGMa relies on ϒ-secretase cleavage of the intracellular portion of Neogenin. ϒ-secretase generates a Neogenin intracellular domain (NeICD) peptide that interacts with LIM-only protein 4 (LMO4) to block axonal growth. We show that distinct axon guidance effects can be mediated by two different RGMa peptides.

## Results

### N-RGMa but not C-RGMa is a repulsive guidance cue

**Figure 1:**
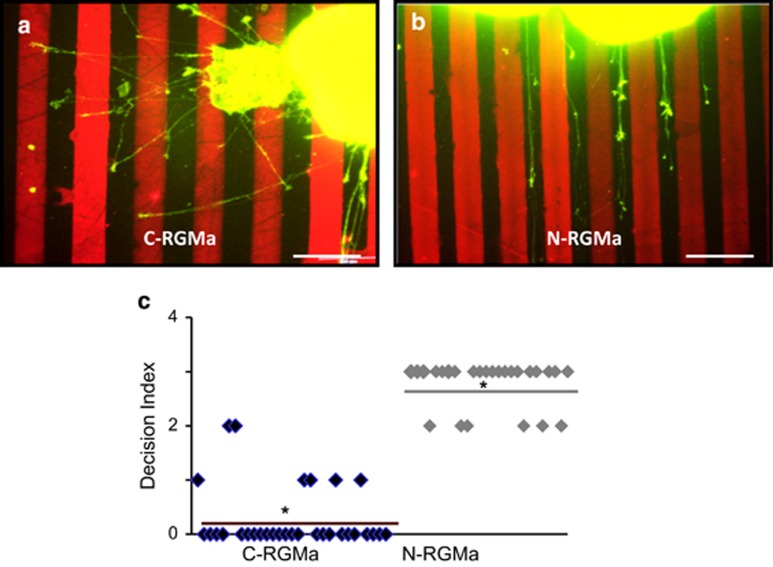
N-RGMa is a repulsive guidance molecule. (**a** and **b**) Temporal retinal explants were cultured on substrates patterned with alternating stripes of laminin and laminin plus RGMa peptides. Axons were stained by using the F-actin stain Alexa Fluor-Phalloidin (Green) and the RGMa stripes were labeled with a red fluorophore. (**a**) When grown on stripes of alternating laminin and laminin plus C-RGMa (10 *μ*g/ml), temporal axons did not show any clear preference. (**b**) On alternating stripes of laminin and laminin plus N-RGMa (10 *μ*g/ml) temporal axons avoided the N-RGMa containing stripes. (**c**) Quantification of the avoidance index showed that N-RGMa (average decision index, 2.8±0.1), but not C-RGMa (average decision index, 0.2±0.1), had a clear repulsive guidance effect on growing axons. *P<0.05

Because C- and N-RGMa interact with the same FNIII (3–4) domain of Neogenin to inhibit axonal outgrowth, it was assumed that these peptides activate the same intracellular pathway.^10^ To address the possibility that this may not be the case, we performed a stripe assay in which Neogenin expressing axons from the temporal part of the retina were confronted with alternating stripes containing laminin or laminin+RGMa proteins (Figures 1a and b). When confronted with N-RGMa stripes, explants showed a decision index of 2.8±0.1, revealing a strong repulsive guidance effect for this fragment (Figure 1c). C-RGMa had a decision index of only 0.2±0.1, suggesting that it had no guidance effect. This result was unexpected as (i) C-RGMa is regarded as the guidance peptide of RGMa^13^ and (ii) this represents the first example of an inhibitory outgrowth protein that is not a repulsive guidance molecule. Thus, we performed careful controls to ensure that this effect did not result from a coating artifact (Supplementary Figure 1). We cultured RGC explants on a uniform substrate prepared under the same conditions as the stripes. Both N-RGMa and C-RGMa displayed similar properties with outgrowth reduced by ~3-fold compared with laminin alone (Supplementary Figure 1). These results not only reveal that both C-and N-RGMa have different guidance properties, but they also suggest that these two peptides may operate via two distinct intracellular pathways.

### C- and N-RGMa ectopic expression show distinct path-finding defects

**Figure 2:**
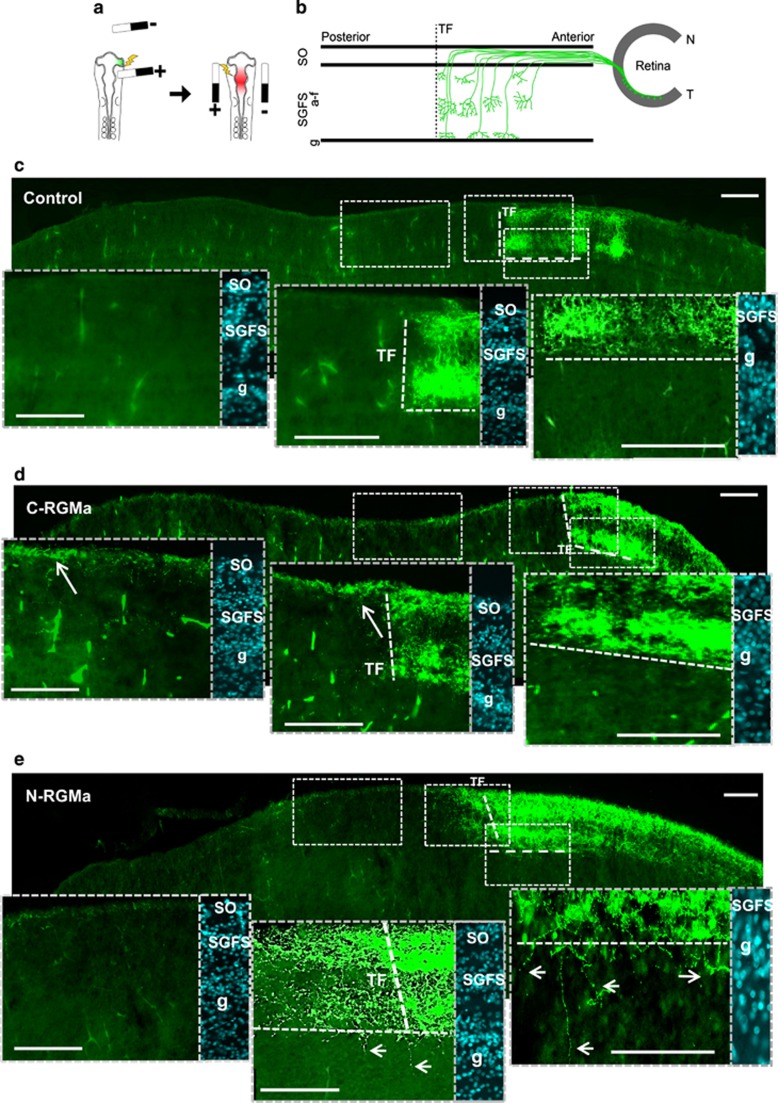
C-RGMa and N-RGMa induce distinct axonal phenotypes. (**a**) Schematic representation of the experimental approach used to study pathfinding. At first electroporation of the optic vesicle is performed to label temporal fibers with GFP. A second electroporation is performed to allow ectopic expression of a plasmid expressing C-RGMa and N-RGMa in the tectum. (**b**) Representation of axonal paths from the retina to the SGFS (**a**–**f**) laminae of the optic tectum. (**c**) In controls, temporal axons populate the SO and SGFS layers and terminate precisely at the predicted terminal front (TF; dotted red line). (**d**) When C-RGMa was overexpressed in the OT, temporal fibers sent overshoots toward the posterior tectum. These overshoots remained restricted to the SO layer (arrow). (**e**) When N-RGMa was overexpressed in the optic tectum, temporal fibers passed the predicted terminal front (dotted line) and sent overshoots toward the posterior tectum. These overshoots established terminal arbors in the SGFS (a–f) layer. Numerous overshoots crossed the SGFS layer g and were also found in deeper tectal layers (arrows). Bar, 100 *μ*m

Until now, it was believed that RGMa peptides had the same function during the establishment of retino-tectal maps. Because the above presented data challenge this assumption we evaluated whether C-RGMa and N-RGMa have the same effect on retino-tectal mapping. To perturb the N- and C-RGMa gradients present in the tectum, we over-expressed each one together with an RFP reporter and animals were killed at E18 (Figures 2a and b). To study axon pathfinding, a GFP-PT2K construct was electroprated in the temporal part of the retina (Supplementary Figures 2–4). This labels temporal axons and leads to the formation of an easily predictable terminal front in the OT.^14^

In tectal whole mounts, we observed that C-RGMa and N-RGMa overexpression induced similar posterior overshoots and aberrant paths (Supplementary Figures 2–4). Controls terminated at the predicted terminal front in the anterior part of the tectum (Figure 2). Next, we investigated axonal growth within tectum laminae. Chick retinal axons first elongate in the SO layer and turn into deeper tectal layers to establish terminal arbors between the layers a and g of the SGFS (Figure 2b). When a control plasmid was electroporated, 10 of the 10 embryos showed a clear border at the expected position within SGFS layers of the tectum (Figure 2c). In contrast, 87.5% of the C-RGMa embryos (7 of 8) displayed overshoots that remained in the superficial SO layer (Figure 2d).

The situation appeared completely different in N-RGMa experiments, where all the embryos (100%, 7 of 7) presented overshoots that were seen in the SGFS layers (Figure 2e). Interestingly, we also observed many fibers that passed the SGFS-g layer, a phenotype never observed in controls (*n*=10). Together, these findings indicate that C-RGMa may be involved in maintaining retinal axons in the superficial SO layer, whereas N-RGMa regulates targeting in deeper tectal layers.

### C-RGMa uses the LARG/Rho/ROCK pathway to block axonal growth

**Figure 3:**
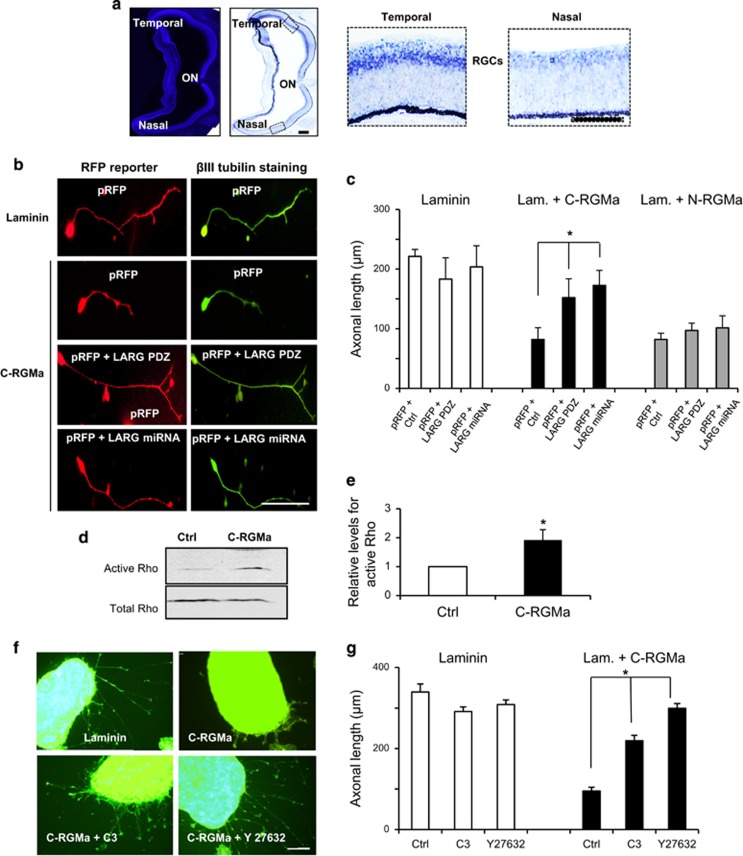
A LARG/Rho/Rock pathway mediates C-RGMa inhibition: (**a**) *In situ* hybridization with a LARG anti-sense probe (LARG-AS) showed LARG expression by RGCs in the chick E8 retina. Negative control, LARG sense (LARG-S). Insets from the temporal and the nasal part of the retina show a low nasal, high temporal expression of LARG. (**b**) RGCs were nucleofected to express an RFP reporter together with either an miRNA or the PDZ domain for LARG. RGC axons that expressed the control pRFP plasmid appeared shorter when cultured on C-RGMa *versus* laminin. The expression of both miRNA for LARG or LARG-PDZ restored outgrowth on C-RGMa. (**c**) Quantification showed that LARG-PDZ and LARG miRNA significantly restored outgrowth on C-RGMa and not on N-RGMa. (**d**) PC12 cells treated with C-RGMa for 30 min showed a stronger signal for active Rho when compared with Control (BSA). (**e**) Quantifications show that C-RGMa significantly increased Rho activation (*P<0.005). (**f**) Temporal retinal explants were cultured on laminin+C-RGMa. Axonal inhibition by C-RGMa was suppressed by both the Rho inhibitor C3-transferase and the Rock inhibitor Y27632. (**g**) C3-transferase and Y27632 significantly reduced C-RGMa inhibition on RGC axons. Bars, 100*μ*m

To further assess the possibility that N- and C-RGMa utilize two separate signaling cascades, we investigated the role of LARG, which interacts with the intracellular part of Neogenin to transduce the inhibition of full-length RGMa.^13^ Based on *in situ* hybridizations, LARG was expressed in a high-temporal low-nasal gradient by RGCs, which is consistent with a role in RGC pathfinding (Figure 3a). Neogenin- expressing temporal RGCs were transfected with the PDZ domain of LARG, which acts as a dominant-negative peptide (Figure 3b), and outgrowth experiments were performed. When cultured on either laminin or laminin+N-RGMa, expression of LARG-PDZ did not affect axonal growth (Figures 3b and c). However, LARG-PDZ restored outgrowth on a C-RGMa containing substrate with the average axonal length increased by ~2-fold (from 82.0±19.6 *μ*m to 152.1.0±19.631.6 *μ*m) compared with control. To confirm this outcome, we generated an miRNA for LARG (Supplementary Figure 5a) which was used to transfect temporal RGCs (Figures 3b and c). When LARG was silenced outgrowth was improved by ~2-fold *versus* control when grown on laminin+C-RGMa (Supplementary Figure 6). Notably, this LARG-miRNA did not suppress the N-RGMa inhibition on temporal RGCs (Figure 3c).

Because LARG is a Rho-GEF that promotes the formation of Rho-GTP (active Rho), we studied Rho activation upon addition of C-RGMa. When C-RGMa was added to PC12 cultures, we observed that Rho-GTP levels increased by ~2-fold compared to control (Figures 3d and e). Consistent with a role of Rho in C-RGMa mediated inhibition, treatment with the Rho inhibitor C3-transferase reduced outgrowth inhibition by C-RGMa (Figures 3f and g). Rho-kinase acts downstream of Rho to mediate axonal inhibition. Therefore, to further assess the pathway involved in C-RGMa inhibition, we cultured RGC explants on C-RGMa in the presence of the Rock inhibitor Y27632. As expected, Y27632 reduced outgrowth inhibition confirming that the involvement of the Rho/Rock pathway in C-RGMa inhibition (Figures 3f and g). These results demonstrated that C-RGMa signals through a LARG/Rho/Rock pathway to block axonal growth. Because LARG neutralization had no effect on outgrowth on N-RGMa, it reinforces the notion that N- and C-RGMa use two different pathways.

### ϒ-secretase releases a NeICD peptide that mediates N-RGMa inhibition

**Figure 4:**
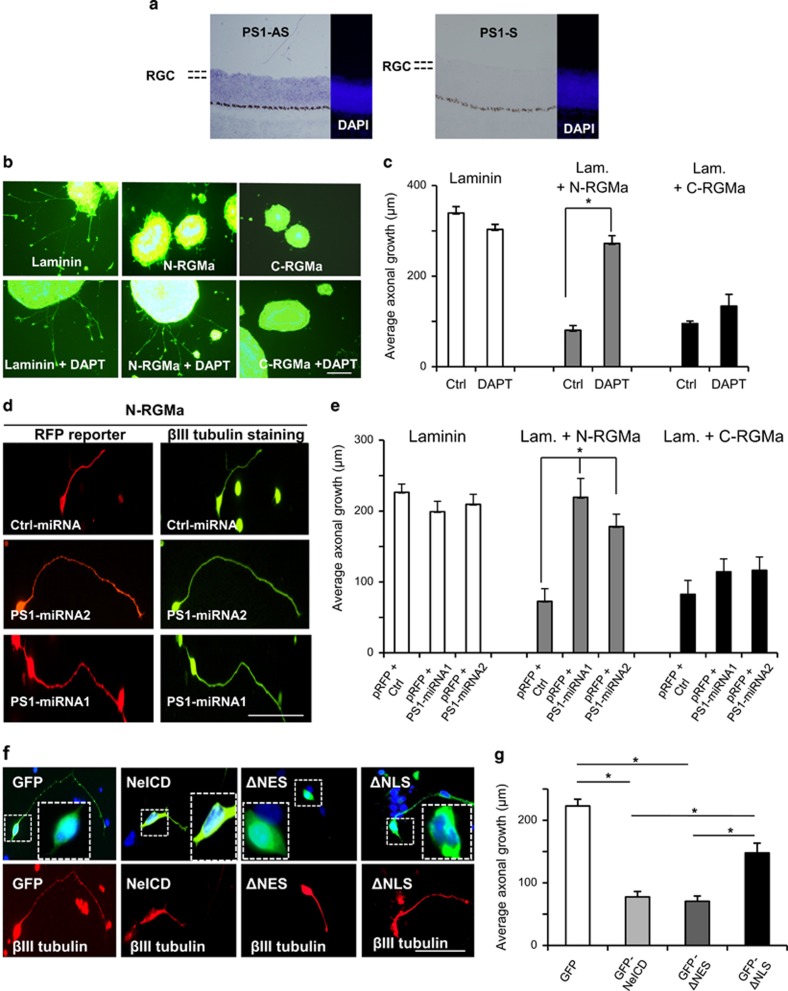
ϒ-secretase and the intracellular domain of Neogenin (NeICD) mediate N-RGMa inhibition. (**a**) *In situ* hybridization with a presenilin-1 anti-sense (PS1-AS) probe demonstrated presenilin-1 expression by RGCs in the chick E8 retina. Negative control, sense (PS1-S). (**b**) RGCs explants were grown on RGMa peptides +/− ϒ-secretase inhibitor (DAPT). (**c**) Quantifications revealed that DAPT significantly suppressed the N-RGMa inhibition on growing axons. DAPT did not affect C-RGMa inhibition. (**d**) Temporal retinal cells were transfected with an RFP reporter together with an miRNA. RGC axons cultured on N-RGMa appeared longer in the presence of presenilin-1 (PS1) miRNAs. (**e**) Quantification showed that PS1-miRNA significantly restored outgrowth on N-RGMa (*P*<0.005). PS1-miRNAs did not affect outgrowth on either laminin or C-RGMa. (**f**) RGCs were transfected with (i) GFP, (ii) NeICD-GFP, or (iii) an NeICD-GFP mutant that lacks the nuclear export signal (ΔNES), or (iv) a mutant that lacks the nuclear localization signal of NeICD (ΔNLS). (**g**) Quantifications revealed that NeICD and ΔNES inhibited axonal growth to the same extent. ΔNLS significantly restored axonal growth when compared with NeICD and ΔNES (*P<0.001). Bars, 100 *μ*m

In cancer cells, Neogenin is proteolytically processed by ϒ-secretase upon binding of soluble full-length RGMa.^15^ To evaluate the involvement of ϒ-secretase in RGMa-mediated axonal inhibition, we studied the expression pattern of Presenilin-1, a member of the ϒ-secretase protein complex, and showed expression by RGCs (Figure 4a). Then, we assessed the effect of DAPT, a ϒ-secretase inhibitor, on RGMa inhibition on retinal explants. DAPT had no effect on temporal explants that grew on laminin alone (Control) and laminin+C-RGMa (Figures 4b and c). Interestingly, DAPT suppressed the N-RGMa effect and axonal growth was increased by over 3-fold compared with vehicle (136.1±8.6 *μ*m and 40.3±5.2 *μ*m, respectively; Figures 4b and c). To confirm these data, we performed silencing experiments using two Presenilin-1 miRNAs (Supplementary Figure 5b). PS1-miRNA-1&2 significantly restored RGC outgrowth on N-RGMa, from 72.7±17.7 *μ*m to 219.4±28.6 *μ*m and 178.1±16.5 *μ*m, respectively (Figures 4d and e). When tested on either Laminin or C-RGMa, these miRNAs did not affect axonal growth (Figures 4d and e; Supplementary Figure 6).

ϒ-secretase induces the release of a NeICD upon binding of RGMa.^15^ To test whether the release of this NeICD domain into the intracellular milieu induces axonal inhibition, we expressed NeICD-GFP into temporal RGC cultures and assessed axonal length. As expected, neurons transfected with NeICD-GFP extended very short axons when compared with GFP-transfected ones (Figures 4f and g). In NeICD-GFP expressing neurons, axons were ~3-fold shorter than those transfected with GFP alone (77.5±9.6 *μ*m *versus* 222.7±10.6 *μ*m). Taken together, these data indicate that Neogenin cleavage by ϒ-secretase generates an NeICD peptide that inhibits axonal growth.

The NeICD contains two domains that regulate its transport in and out of the nucleus.^15^ In HEK293 cells, we have shown that deletion of the nuclear export signal (ΔNES) increases the amount of nuclear NeICD, whereas the deletion of the nuclear localization signal (ΔNLS) reduces NeICD levels in the nucleus.^15^ To address the importance of nuclear localization on NeICD activity, we transfected both ΔNLS and ΔNES in RGCs. Deletion of the nuclear export signal did not restore axonal growth as ΔNES transfection resulted in axonal length similar to the one obtained with NeICD (Figures 4f and g). Interestingly, ectopic expression of ΔNLS significantly increased axonal length (147.7±9.6 *μ*m) when compared with wild-type NeICD (77.5±8.6 *μ*m; Figures 4f and g). Thus, NeICD inhibition relies on its transport to the nucleus.

### The transcription regulator LMO4 is involved in N-RGMa signaling

**Figure 5:**
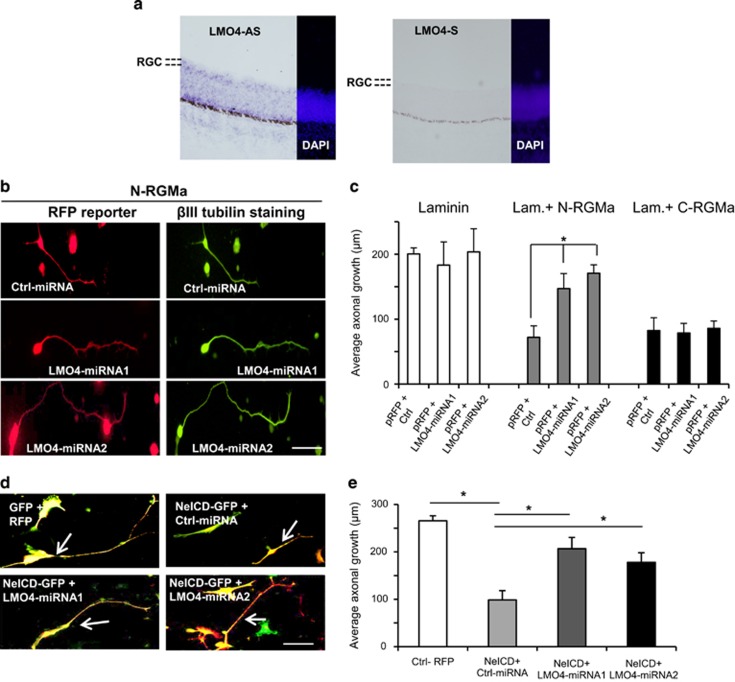
LMO4 mediates N-RGMa inhibition on growing axons. (**a**) *In situ* hybridization with a LMO4 anti-sense (LMO4-AS) probe demonstrated LMO4 is expressed by RGCs in the chick E8 retina. Negative control, sense (LMO4-S). (**b**) Temporal retinal cells were transfected with an RFP reporter together with an miRNA. RGC axons cultured on N-RGMa appeared longer in the presence of LMO4 miRNAs. (**c**) Quantification showed that LMO4-miRNAs significantly restored outgrowth on N-RGMa. Outgrowth on Laminin or C-RGMa was not affected by LMO4-miRNAs (*P*<0.005). (**d**) RGCs were transfected with a GFP reporter together with control miRNA-RFP, or NeICD-GFP together with miRNAs-RFP. NeICD-GFP transfected axons appeared shorter than GFP. Axonal length in NeICD axons was increased by the presence of LMO4-miRNAs. (**e**) Quantifications showed that LMO4-miRNAs significantly restored axonal growth in NeICD transfected cells. *P<0.05

The above presented data indicate that NeICD nuclear import is involved in the N-RGMa inhibition. The NeICD domain of Neogenin interacts with the transcription regulator LMO4.^15, 16^ Thus, it is possible that NeICD interacts with LMO4 to regulate the expression of proteins that block axonal growth. To address this hypothesis, we first studied LMO4 expression in the developing chick eye. *In situ* hybridization showed that LMO4 is expressed by E8 RGCs (Figure 5a).

To evaluate the role of LMO4 in N-RGMa inhibition, we developed two miRNAs for LMO4 (Supplementary Figure 5c). When RGCs were cultured on N-RGMa, both LMO4-miRNAs increased axonal length by >2-fold compared with control (from 72.1±17.6 *μ*m to 147.1±23.1 *μ*m for LMO4-miRNA1 and 170.8±12.9 *μ*m for LMO4-miRNA2; Figures 5b and c). These miRNAs did not affect outgrowth on either laminin or C-RGMa. Next, we studied the effects of these LMO4-miRNAs on NeICD-mediated inhibition. In NeICD-transfected cells, co-transfection with LMO4-miRNA1 increased outgrowth by ~2-fold compared with control miRNA (from 98.6±19.6 *μ*m to 206.7±23.8 *μ*m; Figures 5d and e). Similarly, LMO4-miRNA2 increased axonal length to 178.7±20.1 *μ*m. Together with the ΔNLS data, these results suggest that LMO4 regulated protein expression mediates the N-RGMa inhibition on growing axons.

### LARG-PDZ and NeICD induce defects resembling that of C- and N-RGMa, respectively

Having shown that N- and C-RGMa activate two distinct pathways to inhibit axonal growth, we investigated whether inhibiting these pathways would reproduce the *in vivo* phenotypes observed with overexpression of these two peptides.

**Figure 6:**
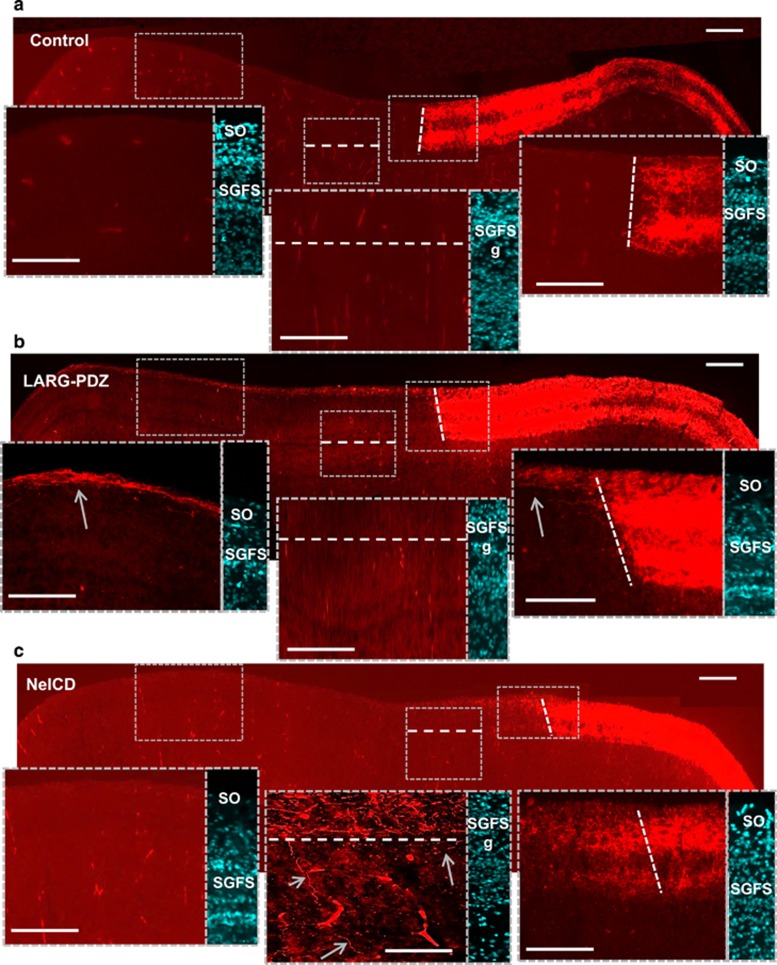
LARG and NeICD induce distinct axonal phenotypes. Electroporation of two plasmids was performed in the eye. (**a**) Control experiment. (**b**) When LARG-PDZ was expressed in the temporal eye, temporal fibers sent overshoots in the SO laminae toward the posterior tectum. This phenotype is similar to the one obtained with C-RGMa, and is consistent with a role of LARG in mediating C-RGMa activity on RGCs. (**c**) When NeICD was expressed in the temporal eye, temporal fibers displayed phenotypes that were similar to the one obtained with N-RGMa. Axons crossed the predicted terminal front (dotted line) and the SGFS layer g (arrows). Bar, 100 *μ*m

To study the implication of the C-RGMa/LARG pathway, we expressed LARG-PDZ in temporal fibers. An RFP reporter was co-expressed to study axonal paths. In control experiments all fibers terminated at the predicated terminal front (Figure 6a; Supplementary Figure 7). Similar to the phenotype observed with C-RGMa, 87.5% (7 of 8) of the embryos expressing LARG-PDZ displayed overshoots that did not enter the SGFS and localized to the SO layer (Figure 6b; Supplementary Figure 8). The fact that both C-RGMa and LARG-PDZ induced the ectopic projections in the SO seems contradictory as one activates the C-RGMa/LARG pathway whereas the other inhibits it. However, during the formation of anterio-posterior maps, overexpression and silencing of RGMa induce the same anterio-posterior mistakes.^17^ While these experiments do not directly show that C-RGMa signaling through LARG regulates axonal paths, they are consistent with that model and provide the first *in vivo* evidence that LARG has a role in map formation. Together, these results indicate that the C-RGMa/LARG pathway is involved in maintaining axonal projections within the most superficial SO layer.

Because ϒ-Secretase is involved in many biological events, we performed ectopic expression of NeICD, which results from the specific action of ϒ-Secretase on Neogenin. As expected, embryos that received NeICD presented a phenotype that appeared similar to the one observed with N-RGMa (Figure 6c; Supplementary Figure 9). In 73% of the embryos (8 of 11), anterio-posterior overshoots were seen in the SGFS layers and many fibers were observed beyond the SGFS layer g. Taken together, this indicates that the N-RGMa pathway is not only involved in anterio-posterior targeting, but also regulates deep layer projections. These results also reveal that the C-RGMa/LARG and N-RGMa/ ϒ-Secretase pathways have distinct roles during the establishment of retino-tectal mapping.

### C-RGMa/LARG inhibits deep layer targeting

**Figure 7:**
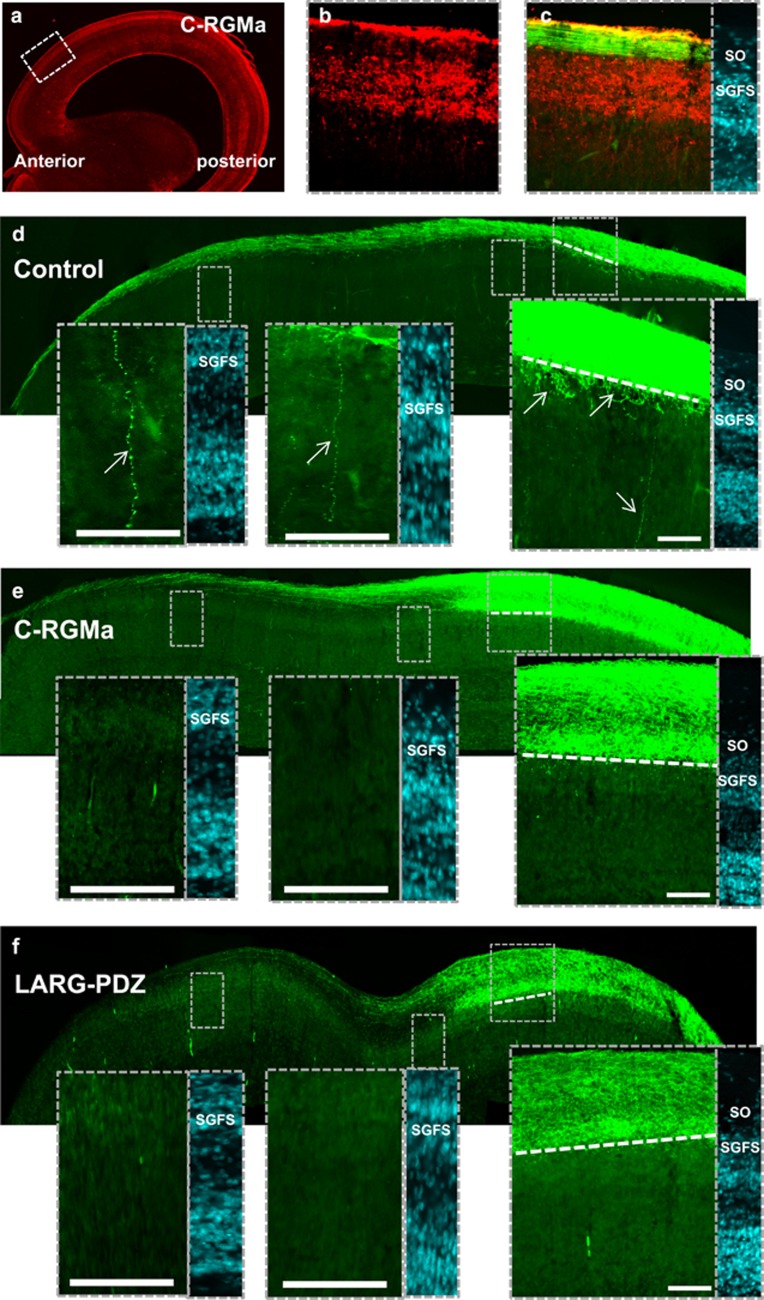
C-RGMa restricts axonal projections to superficial tectal layers. (**a**) Sections of the E12 chick tectum were stained with an antibody for C-RGMa. (**b**) Magnification of the section presented in (**a**) reveals that C-RGMa is expressed in the SGFS but not in the SO layer of the tectum. Inset on the right presents a DAPI staining of the tectal layers. (**c**) Retinal fibers were labeled by electroporation of a GFP expressing plasmid in the E2 chick eye. In the E12 tectum, GFP-positive axons do not enter the C-RGMa positive areas. (**d**–**f**) The E2 eye was electroporated with an GFP expressing constructs and chick were killed at E12. (**d**) When a control plasmid was electroporated, temporal fibers sent overshoot in the SGFS toward deep tectal layers (arrows). (**e**) In contrast, when C-RGMa was expressed in the temporal eye, we did not observe overshoots within deeper tectal layers. (**f**) A similar phenotype was obtained in LARG-PDZ electroporated animals, where no overshoots were observed toward deeper tectal layers. The predicted terminal front is represented by a dotted line. DAPI staining of the tectum are presented as inserts. Bar, 100 *μ*m

N-RGMa and NeICD ectopic expression induced aberrant projections beyond (i) the predicted terminal front and (ii) the layer g of the SGFS. These ectopic projections formed terminal arbors, which is consistent with a role for this pathway in anterio-posterior mapping (Figure 2). The situation appeared different when C-RGMa and LARG-PDZ were overexpressed, and ectopic projections remained confined to the SO layer. Similarly to mammals, chick retino-tectal projections overshoot their terminal destination before being corrected by pruning. Thus, we considered the possibility that the overshoots observed at E18 in the SO layer of C-RGMa-treated animals may result from an altered pruning process. To determine the role of C-RGMa at earlier stages, we stained the E12 tectum with antibodies for C-RGMa and full-length RGMa (Figure 7; Supplementary Figure 10). Interestingly, C-RGMa was found in the SGFS but not in the SO layer of the tectum. Staining for full-length RGMa displayed the same pattern suggesting that N- and C-RGMa are present in the same tissues. Furthermore, axonal labeling revealed that retinal projections avoid the C-RGMa expressing SGFS layer of the tectum. Because this suggested that C-RGMa is involved in preventing axonal projections toward deeper tectal layers, we evaluated whether C-RGMa ectopic expression altered layer projections. When C-RGMa was co-electroporated with an RFP reporter, the red florescence mostly localized with the SGFS where we hypothesized C-RGMa acts as a barrier (Supplementary Figure 11). In controls, 90% (9 of 10) of the embryos displayed long ectopic projections within deep tectal layers. Moreover, the border between the SO and the SGFS appeared diffuse with numerous short projections within the SGFS. In contrast, 82% (9 of 11) of the C-RGMa electroporated animals did not display any long projection in deeper tectal layer. Furthermore, the border between the SO and the SGFS appeared very sharp with almost no projections in the SGFS. In both control and C-RGMa experiments, we observed ectopic projections beyond the predicted terminal front.

Next, we studied the effect of LARG-PDZ expression in retinal axons. In the E12 tectum, axonal projections appeared similar to the one observed with C-RGMa ectopic expression and 90% of the embryos (9 of 10) did not present any deep layer projections (Figure 7; Supplementary Figure 12). Also, similar to C-RGMa, the border between the SO and the SGFS appeared very sharp. Together, these data suggest that the C-RGMa/LARG pathway prevents ectopic projections toward deeper tectal layers.

## Discussion

Here we uncover novel insights on how developing retinal projections target appropriate tectal layers. We show that two RGMa peptides use two distinct intracellular pathways to guide retinal axons toward appropriate target layers. Thus, modulation of these pathways results in the dysregulation of distinct aspects of tectal layer innervation.

The phenotype observed with C-RGMa, which induced overshoots in the SO of E18 embryos may result from a delayed axonal pruning since axons overshoot the terminal front during development in the chick. Another phenotype observed with C-RGMa, which prevented invasion of the SGFS in the E12 embryo, suggests that it is involved in preventing overshoots toward deeper tectal layers. This uncovers a yet unappreciated role for RGMa proteins, which are not only involved in anterio-posterior extension but may also prevent projections into deeper layers. This action on growing axons contrasts with the one observed with N-RGMa ectopic expression, for which anterio-posterior overshoots populate the SGFS lamina. The N-RGMa phenotype resembles the one observed with ephrin transgenic animals, in which axons populate the SGFS layer.^18^ Therefore, it will be interesting to investigate whether other guidance molecules such as Wnts and ephrins are involved in regulating layer targeting.

Interestingly, N-RGMa induced overshoots beyond the SGFS-g layer indicting that it is also involved in deep laminae projection. Although inhibition of either C-RGMa or N-RGMa downstream signaling cascade recapitulated the phenotype observed with overexpression of either C-RGMa or N-RGMa, respectively, it is still possible that the effects obtained with one peptide result from the neutralization of the other. Indeed, N- and C-RGMa compete for binding to Neogenin;^10^ thus, overexpression of N-RGMa may inhibit the C-RGMa pathway thereby inducing the projections beyond SGFS-g at E18.

RGC axons target a specific subset of morphologically and molecularly distinct layers of the optic tectum.^19^ Recent evidence suggests that retino-recipient tectal layers receive their input from specific subtypes of RGCs.^20, 21^ For instance, in the developing mouse, tOFF-*α*RGCs project exclusively to the superior colliculus and dorsal lateral geniculate nucleus, and are restricted to specific laminar depth within each one of these targets.^22^ The present study does not allow to address the role of C-RGMa *versus* N-RGMa on various RGC populations as these could not be distinguished through our labeling technique. Thus, an alternative explanation for the different layer-targeting phenotypes observed with both proteins could be that C-RGMa and N-RGMa act on different RGC subpopulations. For instance, because C-RGMa prevents layer projections beyond the layer g of the SGFS, it is plausible that it will only act on RGCs that normally project into the layer f of the SGFS.

Studies in mice, zebra-fish and the chick revealed that layer-specific axonal targeting varies among species. In zebra fish, axons enter the tectum from the rostral pole and directly target the sublaminae in which they will establish terminal arbors. In the chick, RGC axons enter the tectum through the SO before diving into one of the three laminae (SGFS;b,d,f), where they establish terminal connections.^23^ Similarly, mouse RGC axons enter the tectum before establishing arbors in the SO or turning toward the lower or the upper half of the stratum griseum superficiale. There is an unfortunate dearth of knowledge concerning the nature of the proteins that regulate layer projections, which is largely due to a heretofore lack of intensive study when compared with other aspects of retino-tectal projection. Although the molecular determinants for layer targeting are conserved among species, there may be some differences. Thus, RGMa is involved in anterio-posterior projection in the chick but not in the mouse.^24^ Since RGMa is expressed in the mouse superior colliculus,^24^ it most likely influences growing RGCs, hence, it will be interesting to study whether RGMa regulates murine layer targeting.

If C-RGMa prevents deep layer targeting, then silencing of its downstream effector LARG should promote deep projections. However, LARG silencing appeared to prevent the formation of deeper projections, which may appear to be in contradiction with a repellent activity of C-RGMa. This is not necessarily the case, as it has previously been shown that either ectopic overexpression or silencing of repulsive guidance proteins in the tectum often induces identical phenotypes. For instance, experiments from Matsunaga *et al.*^17^ show that ectopic expression or silencing of RGMa in the tectum both induced temporal fiber overshooting. Similarly, ectopic expression of the repulsive ephrinA5 induces overshooting of temporal fibers that are similar to the one observed with downregulation of this protein.^25^ Thus, silencing or activating one pathway may lead to the same phenotype. LARG and LMO4 mediate the action of full-length RGMa on growing axons.^5, 15^ Here we show that they are part of two separate pathways that mediate different axonal responses. While LARG activates Rho to inhibit outgrowth, LMO4 most likely does so by regulating gene expression. Finally, our findings reveal that ϒ-secretase regulates the action of Neogenin on growing axons. ϒ-secretase cleaves the intracellular domain of DCC thereby coordinating the interplay between Netrin/DCC and slit/Robo signaling. However, unlike the NeICD, the DCC-intracellular domain does not regulate axonal growth.^26^ In contrast, cleavage by ϒ-secretase of the intracellular domain of DCC terminates the action of this receptor on growing neurons. Thus, our work uncovers another layer of ϒ-secretase action during CNS development.

## Materials and Methods

### Cloning, expression, and purification

RGMa peptides were cloned in pSectag2B vector (Invitrogen, Karlsruhe, Germany) with an N-terminal His-tag. They were then transfected into HEK-293 cells for protein production. Soluble proteins were purified using Ni-NTA agarose (Invitrogen), and dialyzed against PBS.

### Retinal explants outgrowth/stripe assay

Glass coverslips were coated with 10 *μ*g/ml Poly-L-Lysine, treated with Laminin (10 *μ*g/ml), +/− RGMa proteins (10 *μ*g/ml). Alternatively, different concentrations of soluble proteins mixed with Laminin (10 *μ*g/ml) were added to the coverslips and incubated for 3 h at RT. Explants from the temporal retina were then added to protein-coated surfaces in DMEM F-12 media (2% chick serum, 10% FBS) and incubated (37 °C, 5% CO_2_) for 18 h. Explants were fixed in 4%PFA, permeablized with 0.1% Triton X-100, stained with Alexa488-fluor-phalloidin and viewed under a fluorescence microscope (Zeiss, Toronto, ON, Canada). The number and length of fibers were then quantified using Image Pro 5.0 (Media Cybernetics, Rockville, MD, USA). Only explants that displayed growth were considered.

For protein stripe assays, ethanol-washed coverslips were incubated in 10 *μ*g/ml poly-L-lysine (Sigma, Oakville, ON, Canada) in water for 2 h at room temperature. Coverslips were rinsed several times with water, air dried, and inverted onto a silicon matrix obtained from Friedrich Bonhoeffer (Max Planck Institute, Tuebingen, Germany). A mix of purified RGMa proteins and laminin (10 *μ*g/ml; Invitrogen) in phosphate-buffered saline (PBS) was injected into the matrix channels and incubated for 2 h at room temperature. After rinsing the channels by injecting PBS, the coverslips were removed and rinsed in PBS. They were then coated with 20 *μ*g/ml laminin in PBS and incubated for 3 h at room temperature. In the next step, the coverslips were washed with PBS, placed in the appropriate culture medium and retinal stripes were transferred to the coverslips and cultured for 18 h.

### Dissociated retinal ganglion cell preparation and nucleofection

Dissociated Retinal ganglion cells were prepared from E7 chick embryos. Retinas were dissected out in HBSS, trypsinized and cultured in DMEM/F12 media with 10% calf serum and N2 supplement. The cells were cotransfected with NeICD and control miRNA, or NeICD and miRNA constructs targeting LMO4 and LARG and LARG pdz using nuclefector kit (Amaxa Chicken Neuron Nucleofector kit; Lonza, Allendale, NJ, USA). The cells were plated (500,000 cells/well) on coverslips coated with Laminin (Millipore, Etobicoke, ON, Canada; 10 *μ*g/ml) or Laminin plus N-RGMa (10 *μ*g), or C-RGMa (10 *μ*g) proteins and grown for 18 h. The cells were fixed in 4% paraformaldehyde and stained with *β*-tubulin antibody (Covance, Burlington, ON, Canada; 1 : 1000) for 1 h at RT, followed by goat anti-mouse secondary antibody (1 : 500) for 1 h at RT. The images of transfected cells were taken using Olympus fluorescence microscope (BX61, Richmond Hill, ON, Canada). The length of axons of transfected cells was measured using cellSens (Olympus, Richmond Hill, ON, Canada). To overexpress NeICD and ΔNLS and ΔNES mutants, cells were nucleofected with constructs and plated on coverslips coated with Laminin.

The miRNA sequences are as follows: LMO4miRNA2-ccagggcaatgtctatcatct; LMO4miRNA3-aagatcggtttcactacatca; PS1miRNA1-actcctggttgtgctttacaaa; PS1miRNA2- ttggtgttgtgggaatgatttg; LARGmi2-cagcattgttgtgctaccttta.

### Retinal fiber tracing

*In ovo* electroporation was performed as described previously.^14, 27^ Briefly fertilized chicken embryos were incubated in humid conditions at 38 °C for 46–54 h to reach stage 11.^28^ To trace RGC fibers by stable gene transfer, EGFP or DS-Red monomer expression cassette flanked by Tol2 transposon (pT2K-CAGGS-EGFP/DS-Red monomer) was transfected to the temporal side of the right optic vesicle with transposase expression vector (pCAGGS-T2TP) (Figure 1).^29^ The mixture of pT2K-CAGGS-EGFP/DS-Red monomer (2 *μ*g/*μ*l) and pCAGGS-T2TP (1 *μ*g/*μ*l) was injected into the lumen of the optic vesicle. Parallel electrodes (0.5 mm in diameter, 1.0 mm in length, 2.0 mm in distance) were placed on the either side of the right optic vesicle perpendicular to the naso-temporal axis of the right optic vesicle. Then, 13 V, 50 ms/s rectangular pulses were charged three times by an electroporator (CUY21, Bex, Tokyo, Japan). NeICD and LARG-PDZ inserted in RCASBP(A) were co-transfected with these tracer vector mixture (Figure 1a). N-RGMa or C-RGMa inserted in pT2K-CAGGS was transfected to the left tectal primordium by second electroporation just after first electroporation (Figure 2a). Parallel electrodes were placed on the either side of the tectal primordium. Then, 13 V, 50 ms/s rectangular pulses were charged three times by an electroporator.^27^ Embryos were re-incubated for 16 days and were killed at E17–E18, when retino-tectal projections have matured in chick embryos.

### Statistical analysis

Quantifications were done for outgrowth assays from at least three independent experiments. Statistical analysis was performed using ANOVA by XLSTAT. Results are expressed as the average±S.E.M.

### *In situ* hybridization

LARG *in situ* hybridization was performed by amplifying the 920–1742 bp region with the primers: Larg-*Kpn*I-For: ggtaccgagattctgagaaagatgctac, Larg-*Hin*dIII-Rev: aagcttgaaatcttccagattcctctga.

Presenilin *in situ* hybridization was performed by amplifying the 662–1480 bp region with the primers: PSEN-*Kpn*I-Forward: gtaccgccatggactacattacag, PSEN-Reverse: ctgatggaatgctagctggt.

LMO4 *in situ* hybridization was performed by amplifying the 86–902 bp region with the primers: LMO4-*Kpn*I-Forward:ggtaccgagcggcgcgaagtgcggccg, LMO4-*Hin*dIII-Reverse: aagcttcatgggatccstctgtgatg.

For *in situ* hybridization on sections, 14*-μ*m-thick sections were cut on a cryostat (Leica, Bannockburn, IL, USA) and processed according to our published protocol.^10^ DIG-labeled antisense RNA probes were generated using the Dig DNA labeling and detection kit from Roche (Laval, QC, Canada). Whole mount *in situ* were processed according to Sections and embryos were developed with 330 *μ*g/ml 4-nitroblue tetrazolium chloride and 160 *μ*g/ml 5-bromo-4-chloro-3-indolyl phosphate in 100 mM Tris-HCl, 5 mM MgCl_2_, pH 9.6.

## Abbreviations

RGM: Repulsive Guidance Molecule
LMO4: LIM-only protein 4
LARG: Leukemia-associated RhoGEF (LARG)
SO: startum opticum
SGFS: stratum griseum et fibrosum superficial
RGC: Retinal Ganglion Cell
NeICD: Neogenin Intracellular Domain
