# Supplementary material for: ϒ-secretase and LARG mediate distinct RGMa activities to control appropriate layer targeting within the optic tectum
Source: Cell Death Differ. 2015 Aug 21;23(3):442–53. doi: 10.1038/cdd.2015.111 (PMC5072438; doi:10.1038/cdd.2015.111)
Supplement: Supplementary Information [file cdd2015111x1.pdf]

Supplementary Figures

Supp. Figure 1

C- and N-RGMa inhibit axonal growth to the same extent: (a-c) Explants were grown on uniform substrate prepared in the same conditions as the stripe substrate (laminin, N- and C-RGMa all at 10 µg/ml on the same poly-L-Lysine pre-coated coverslips). Both C- and N-RGMa inhibited outgrowth when compared to Laminin. d) Quantification shows that N- and C-RGMa significantly inhibited axonal growth to the same extent when compared to laminin (*$P<0.005$)..

Supp. Figure 2

Control for the temporal retinal axon tracing. a) Right eye flat mount. GFP is expressed in the temporal side of the retina with a sharp border at the optic fissure. b) Left tectum whole mount. A sharp border of terminal arbors of GFP labeled axons is observed in the middle of anterio-posteior axis of the tectum (terminal front). c) Higher magnification of the posterior part of the tectum (Posterior box). d) Higher magnification of the anterior part of the tectum (Border box).

Supp. Figure 3

Temporal retinal axon tracing on the C-RGMa misexpressed tectum (E18 tectum). C-RGMa was electroporated together with RFP in the E2 tectum. GFP was electroporated in the E2 temporal retina to follow axonal tracts. a) Picture of the RFP reporter in the C-RGMa/RFP electroporated tectum. b) Right eye flat mount. GFP is expressed in the temporal side of the retina with a sharp border at the optic fissure. c) Left tectum whole mount. d) Higher magnification of the posterior part of the tectum (Posterior box). Overshooting axons exhibit aberrant path. e) Higher

magnification of the anterior part of the tectum (Border box). Numerous axons are overshot towards the posterior tectum.

Supp. Figure 4

Temporal retinal axon tracing on the N-RGMa misexpressed tectum (E18 tectum). N-RGMa was electroporated together with RFP in the E2 tectum. GFP was electroporated in the E2 temporal retina to follow axonal tracts. a) Picture of the RFP reporter in the N-RGMa/RFP electroporated tectum. b) Right eye flat mount. GFP is expressed in the temporal side of the retina with a sharp border at the optic fissure. c) Left tectum whole mount. The terminal front is indistinct. d) Higher magnification of the posterior part of the tectum (Posterior box). e) Higher magnification of the anterior part of the tectum (Border box). Numerous axons are overshot towards the posterior tectum.

Supp. Figure 5

Silencing of LARG, Presenilin and LMO4. To ensure that miRNAs silenced the expression of their target proteins, we cloned chick LARG, Presenilin, and LMO4 as His-tagged proteins and co-expressed them with their respective miRNAs.

a) Recombinant His tagged LARG was expressed in HEK cells in the presence of a Control miRNA or a Larg-miRNA-1. Co-transfection with Larg-miRNA-1 strongly reduced the signal detected with an anti-His antibody.

b) Recombinant His tagged Presenilin 1 was expressed in HEK cells in the presence of a Control miRNA or PS1-miRNAs. Co-transfection with PS1-miRNA-1 and PS1-miRNA-2 strongly reduced the signal detected with an anti-His antibody.

c) Recombinant His tagged LMO4 was expressed in HEK cells in the presence of a Control miRNA or a LMO4-miRNAs. Co-transfection with LMO4-miRNA-1 and LMO4-miRNA-2 strongly reduced the signal detected with an anti-His antibody.

Supp. Figure 6

Assessment of miRNAs on axons growing on laminin. To ensure that miRNAs did not have any general effect on axonal outgrowth, we transfected dissociated RGCs with them and assessed outgrowth on laminin. RGCs were visualized with the neuronal marker for βIII-tubulin (green) and miRNAs co-express an RFP marker (red).

a) None of the tested miRNAs affected outgrowth on laminin.

b) Quantification of axonal growth on laminin did not show any significant effect of the miRNAs

Supp. Figure 7

Control for the temporal retinal axon tracing. a) Right eye flat mount. RFP is expressed in the temporal side of the retina with a sharp border at the optic fissure. b) Left tectum whole mount. A clear terminal front is observed. c) Higher magnification of the posterior part of the tectum (Posterior box). d) Higher magnification of the anterior part of the tectum (Border box).

Supp. Figure 8

Temporal retinal axon tracing for LARG-PDZ misexpressed RGC. a) Right eye flat mount. RFP is expressed in the temporal side of retina with a sharp border at the optic fissure. b) Left tectum whole mount. c) Higher magnification of the posterior part of the tectum (Posterior box).

Overshot axons exhibit straight path. d) Higher magnification of the anterior part of the tectum (Border box). Numerous axons are overshot towards the posterior tectum.

Supp. Figure 9

Temporal retinal axon tracing for NeICD misexpressed RGC. a) Right eye flat mount. RFP is expressed in the temporal side of retina with a sharp border at the optic fissure. b) Left tectal whole mount. c) Higher magnification of the posterior part of the tectum (Posterior Box). Overshot axons exhibit the aberrant path. d) Higher magnification of the anterior part of the tectum (Border box). Numerous axons are overshot towards the posterior tectum.

Supp. Figure 10

Staining of the E12 optic tectum with RGMa antibodies. a) staining of the tectum with a polyclonal goat antibody (R&D AF 2458) raised against full length RGMa. b) Tectum stained with a mouse anti C-RGMa antibody (see Tassew et al., 2009)[12]. c) Merge of the staining presented in a and b.

d-f) Higher magnification of the insets presented in a, b and c.

Supp. Figure 11

Temporal retinal axon tracing on the C-RGMa misexpressed tectum (E12 tectum). C-RGMa was electroporated together with RFP in the E2 tectum. GFP was electroprated in the E2 temporal retina to follow axonal tracts. a) Picture of the RFP reporter in the C-RGMa/RFP electroprated

E12 tectum. b) Picture of the RFP reporter in the C-RGMa/RFP electroprated E12 tectum together with GFP stained Temporal axons. c) Insets presented in a and b are shown.

Supp. Figure 12

C-RGMa restrict axonal projections to superficial tectal layers . a-c) The E2 eye was electroporated with an GFP expressing constructs and chick were sacrificed at E12. a) When a control plasmid was electroporated, temporal fibers sent overshoot in the SGFS towards deep tectal layers (arrows). b) In contrast, when C-RGMa was expressed in the optic tectum, we did not observe overshoots within deeper tectal layers. c) A similar phenotype was obtained when LARG-PDZ was electroporated in the eye, where no overshoots were observed towards deeper tectal layers. The predicted terminal front is represented by a dotted line. DAPI staining of the tectum are presented as inserts. Bar, 100µm.

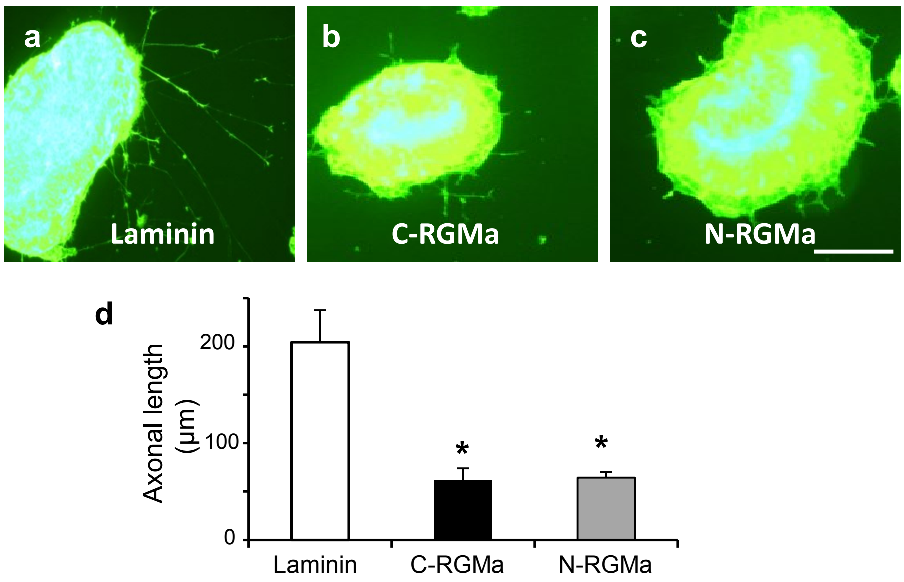

Supp. Figure 1

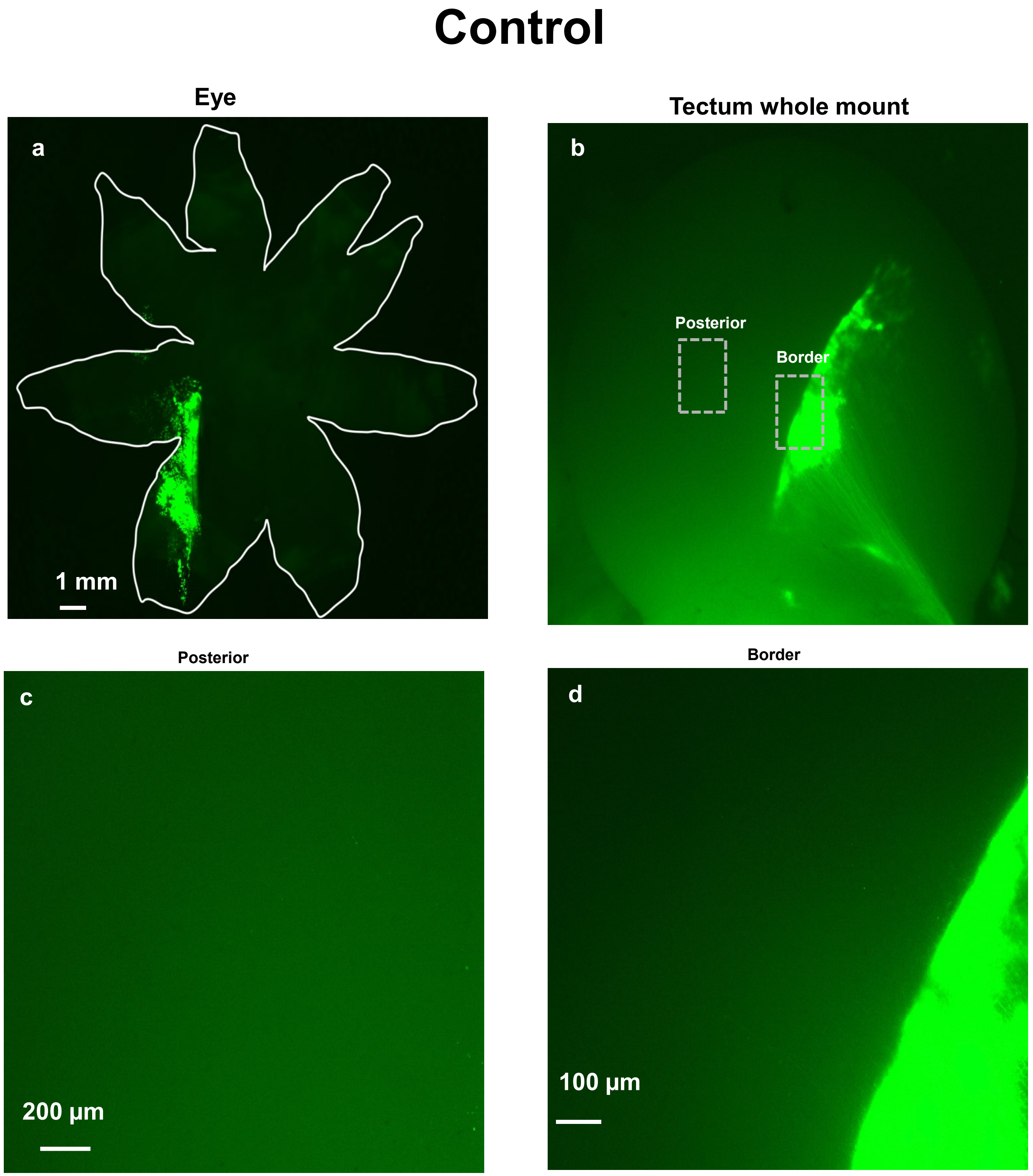

Supp. Figure 2

C-RGMa misexpression

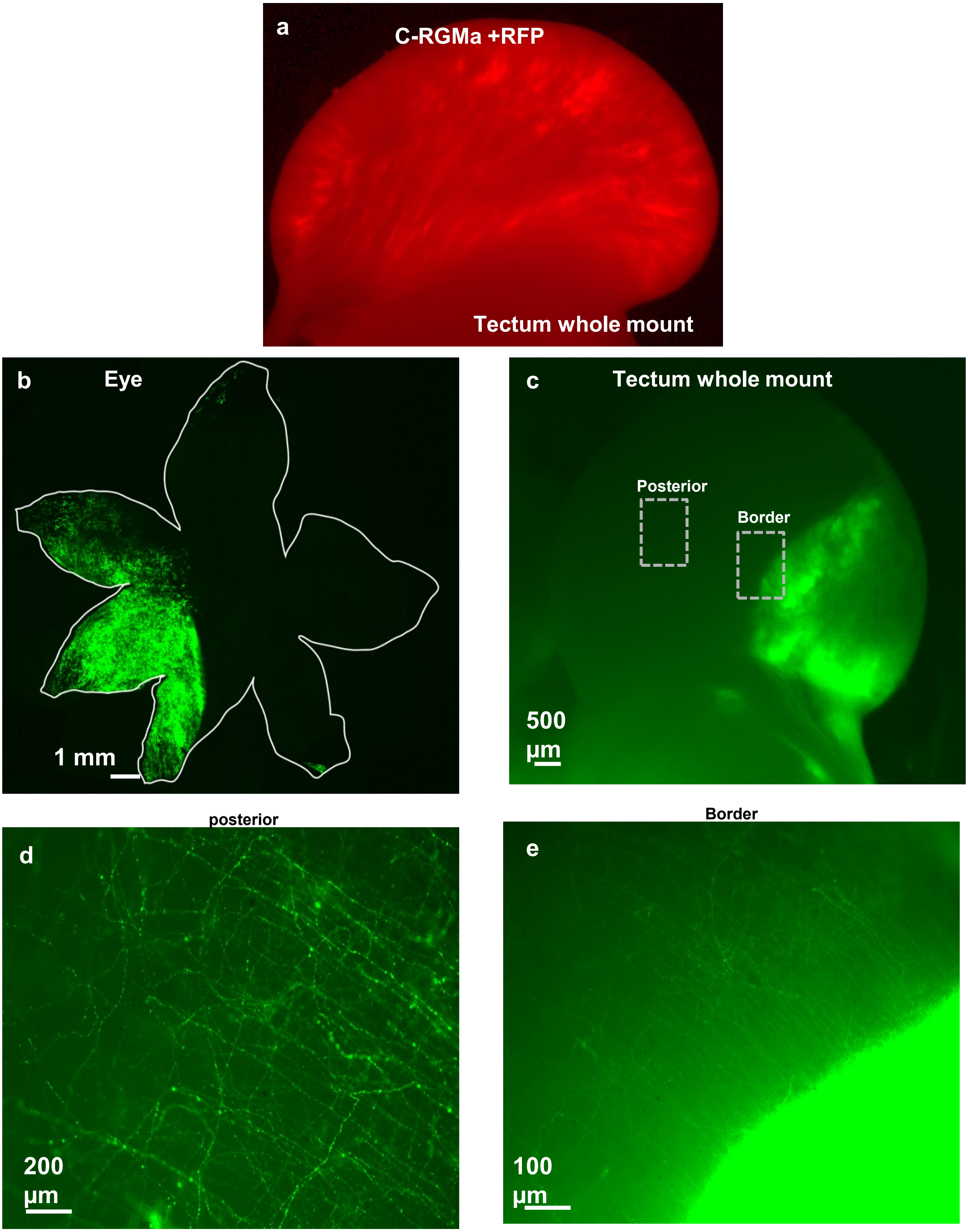

Supp. Figure 3

N-RGMa misexpression

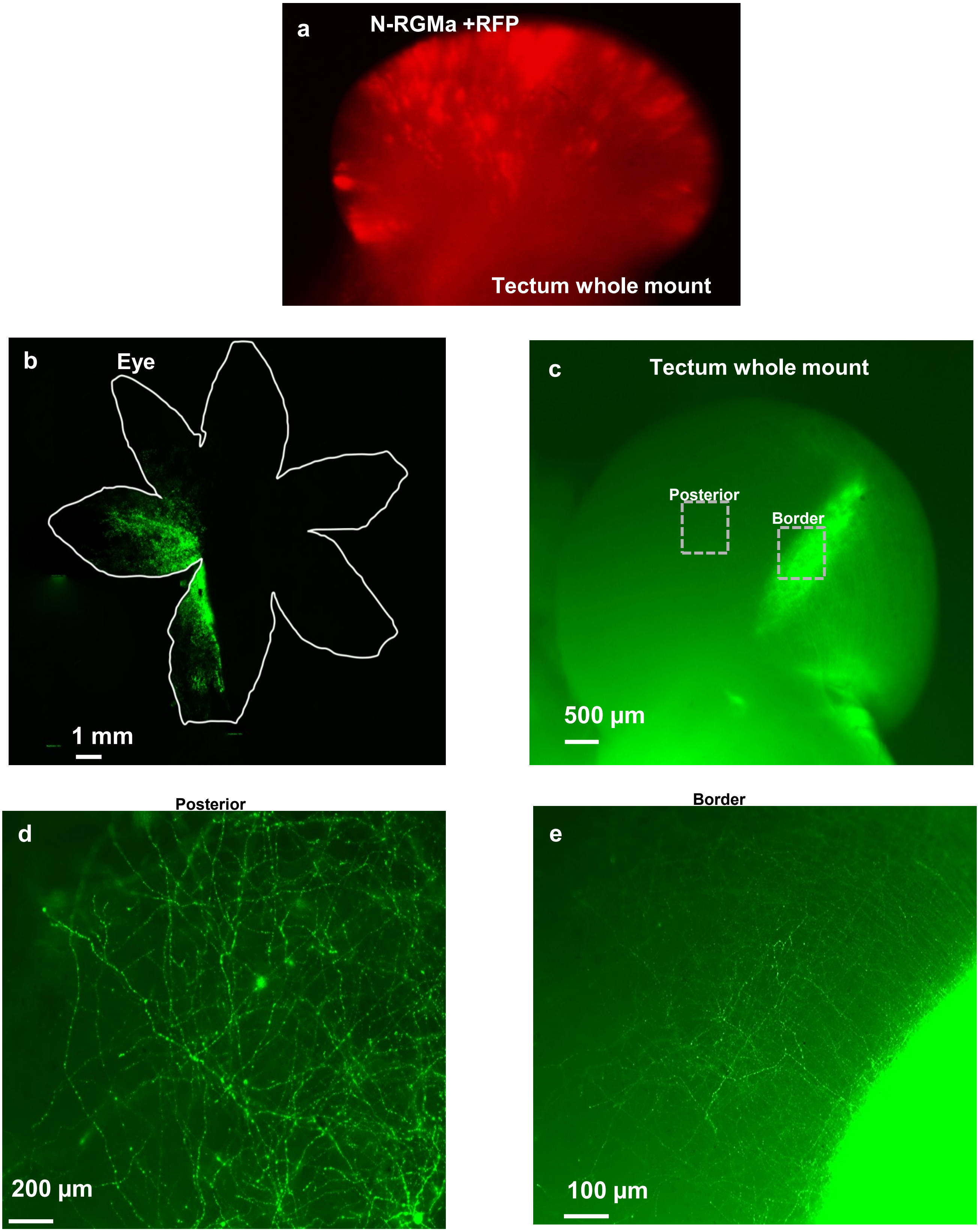

Supp. Figure 4

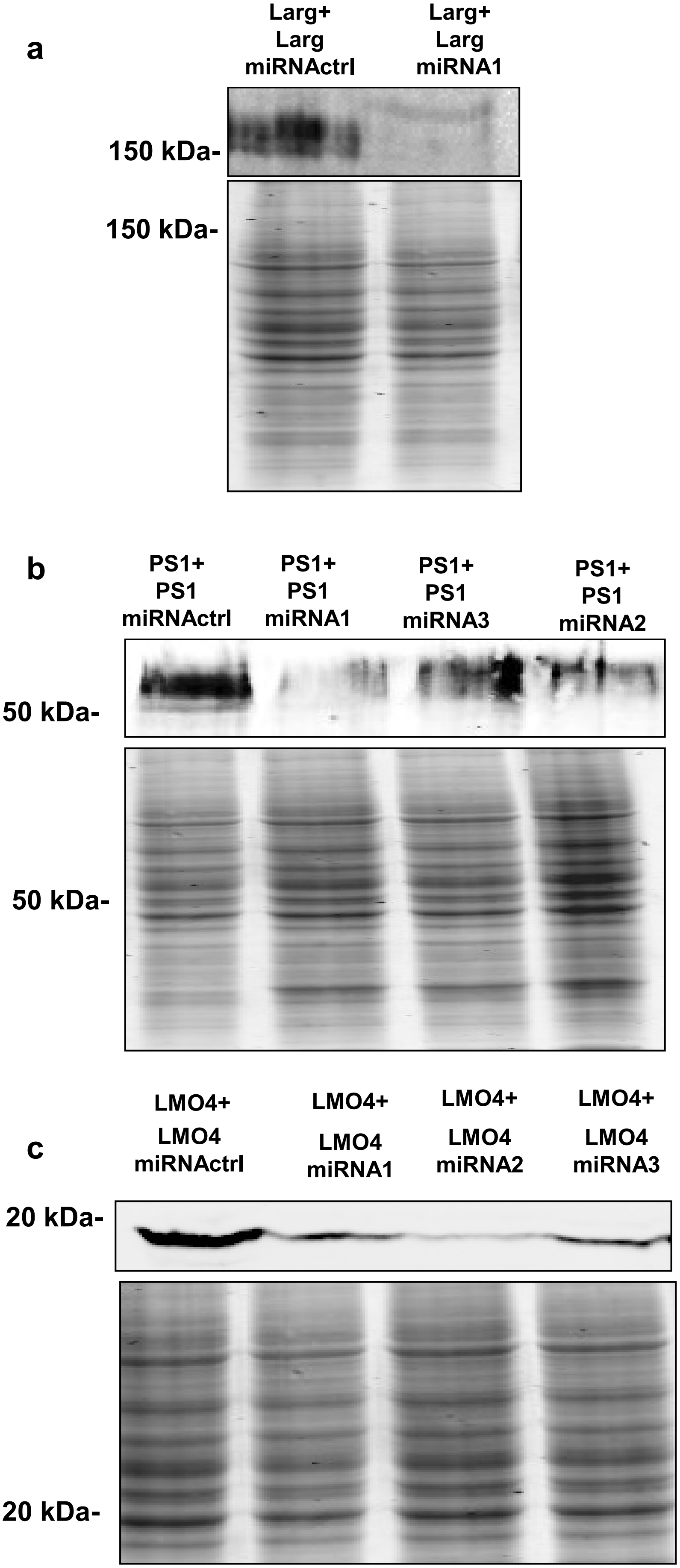

Supp. Figure 5

a

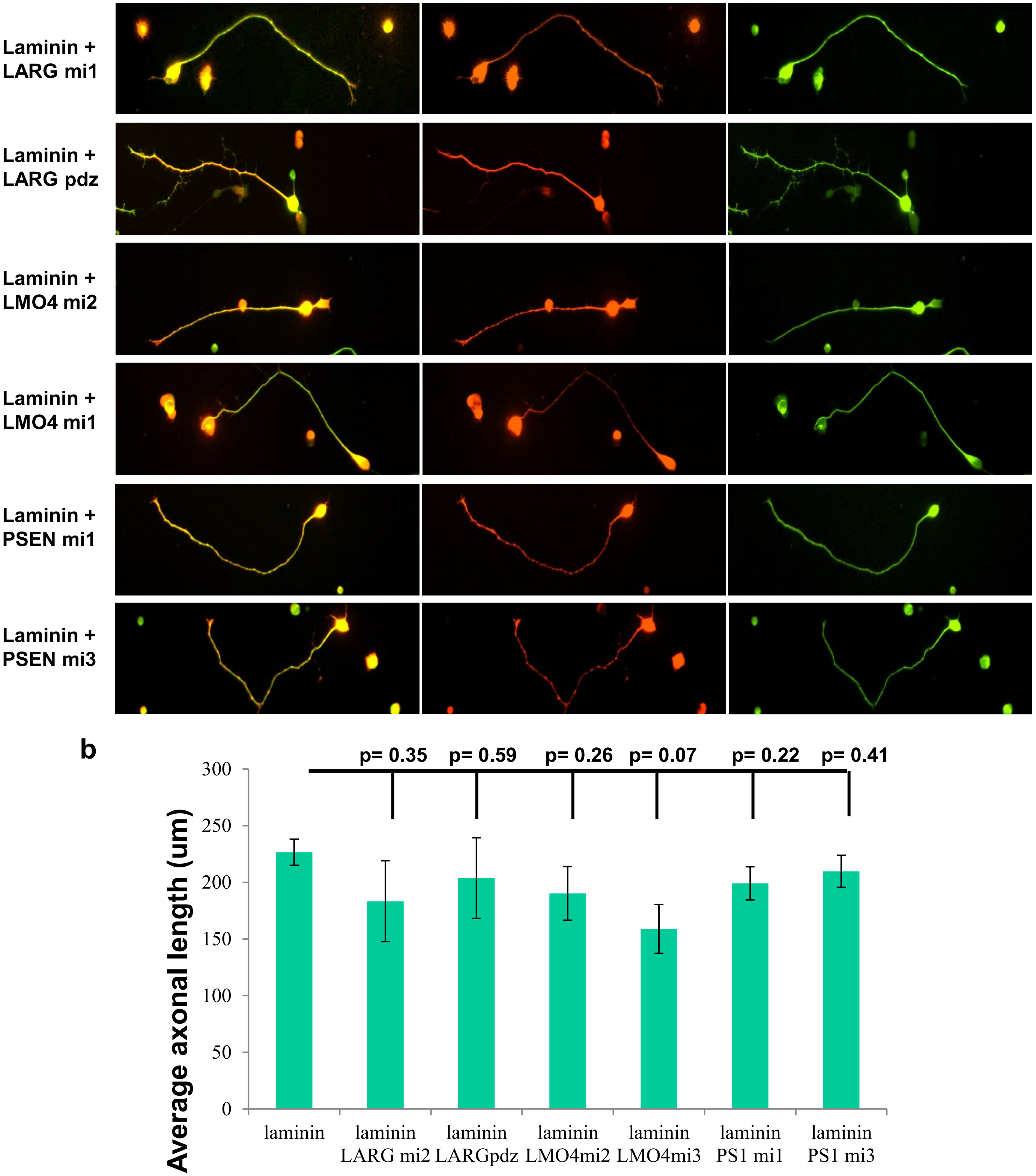

Supp. Figure 6

Control

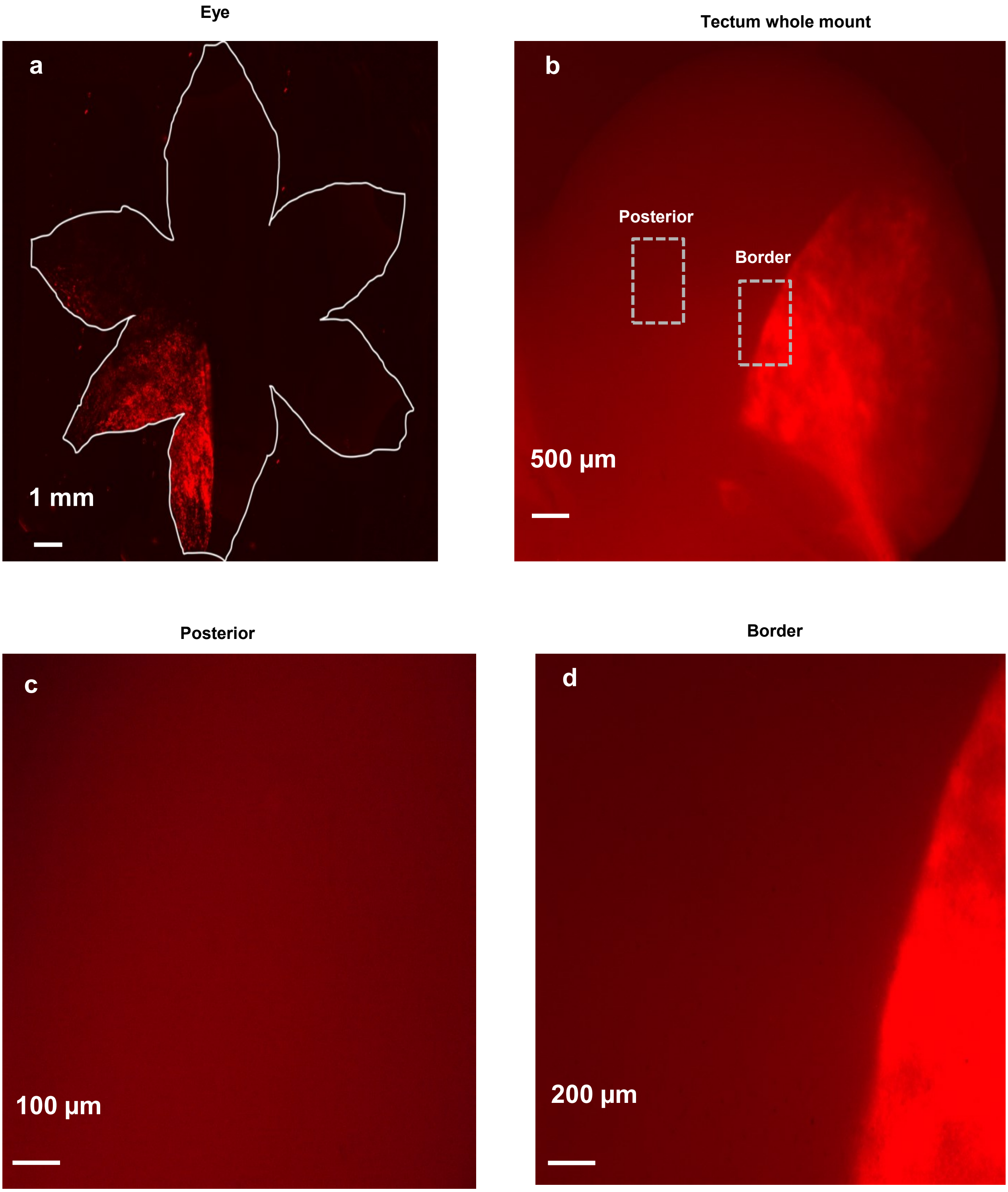

Supp. Figure 7

LARG pdz

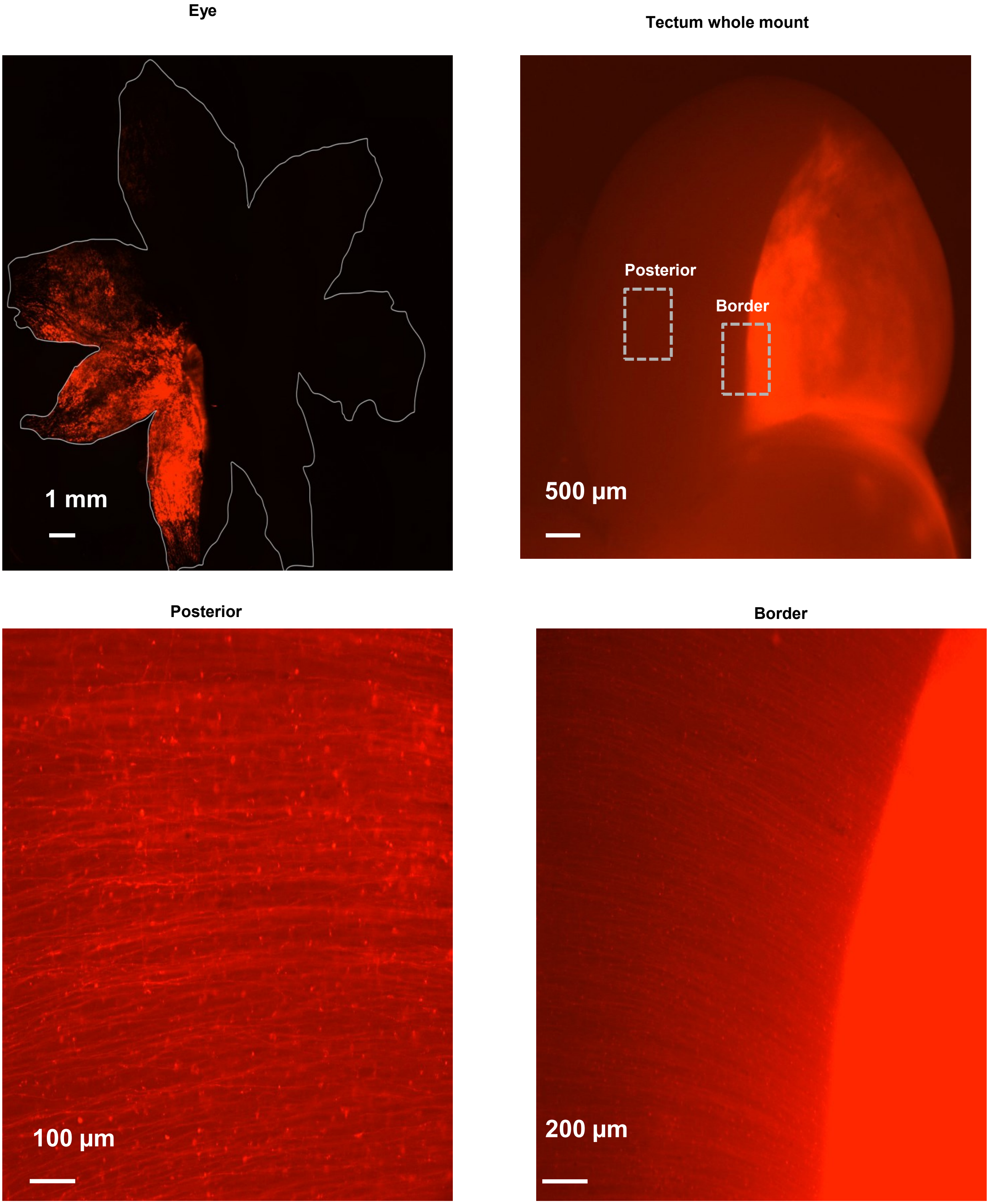

Supp. Figure 8

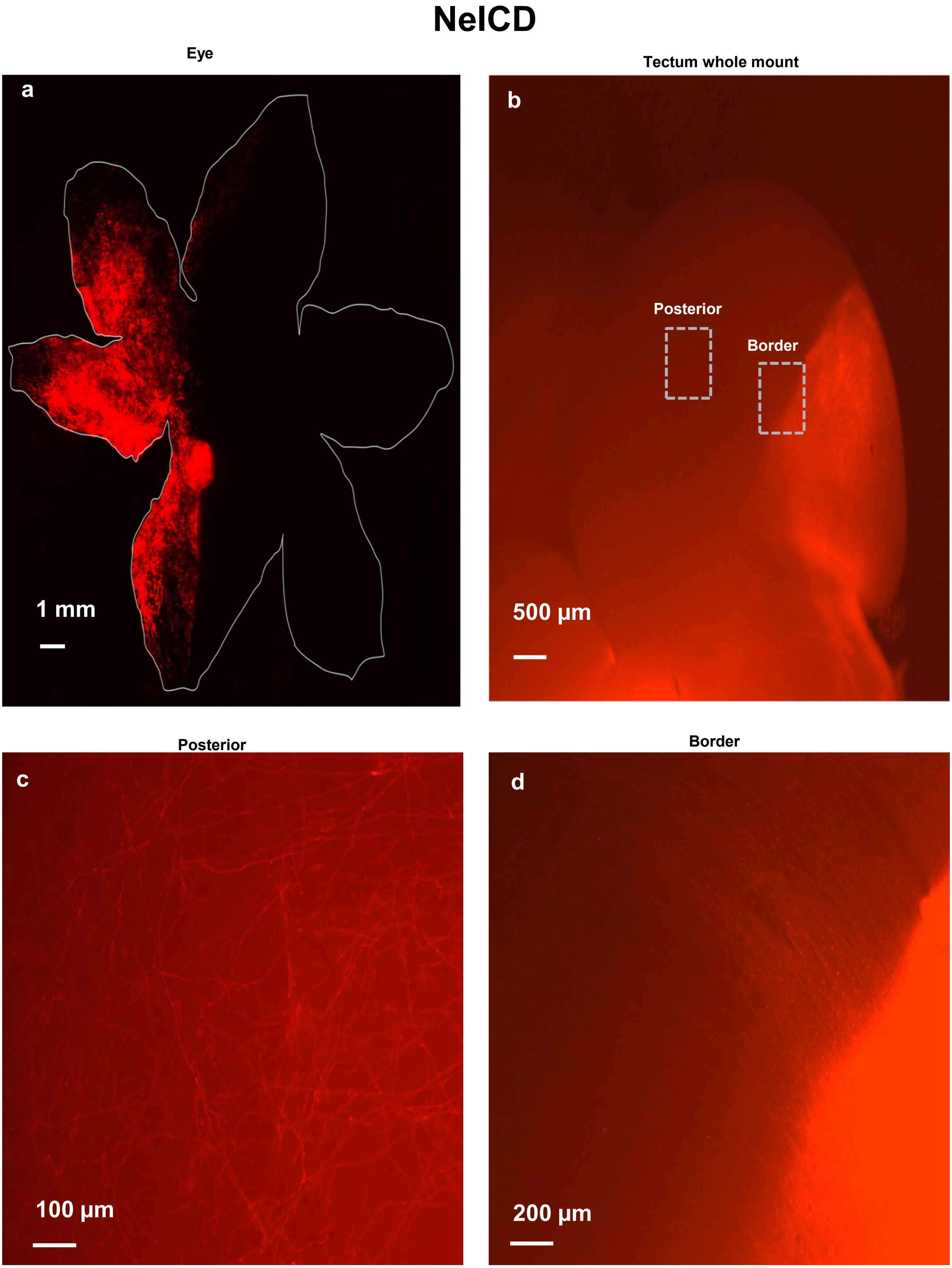

Supp. Figure 9

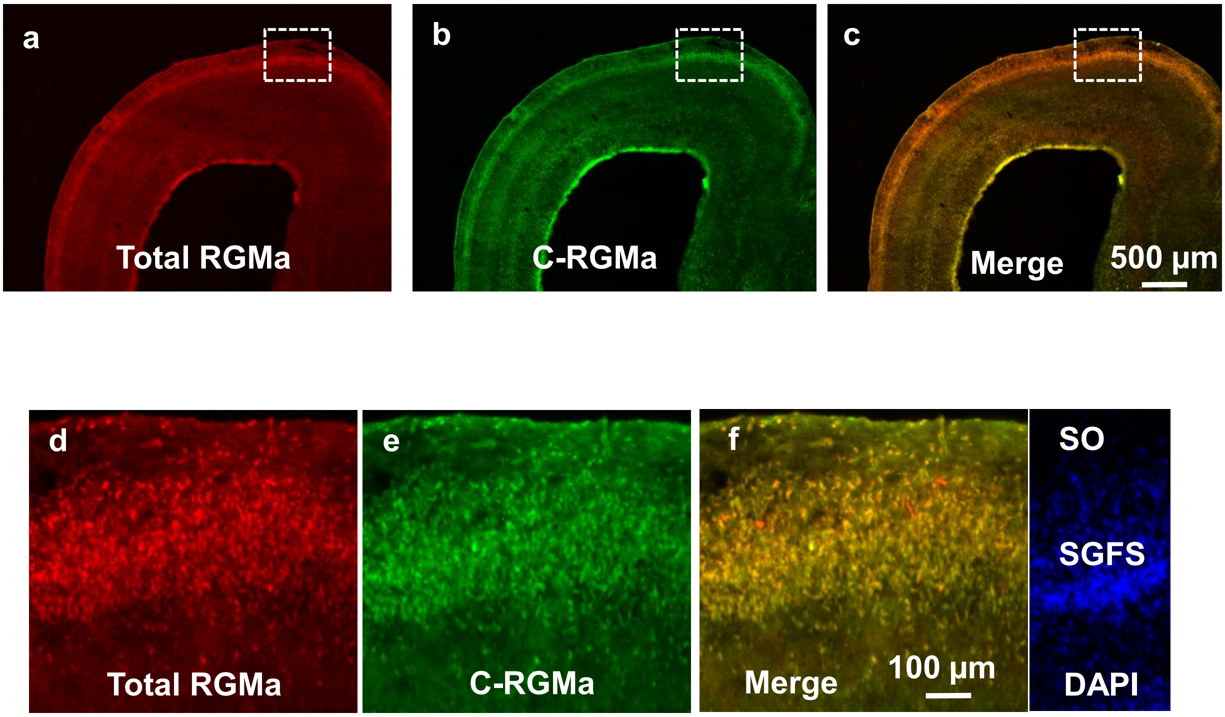

Supp. Figure 10

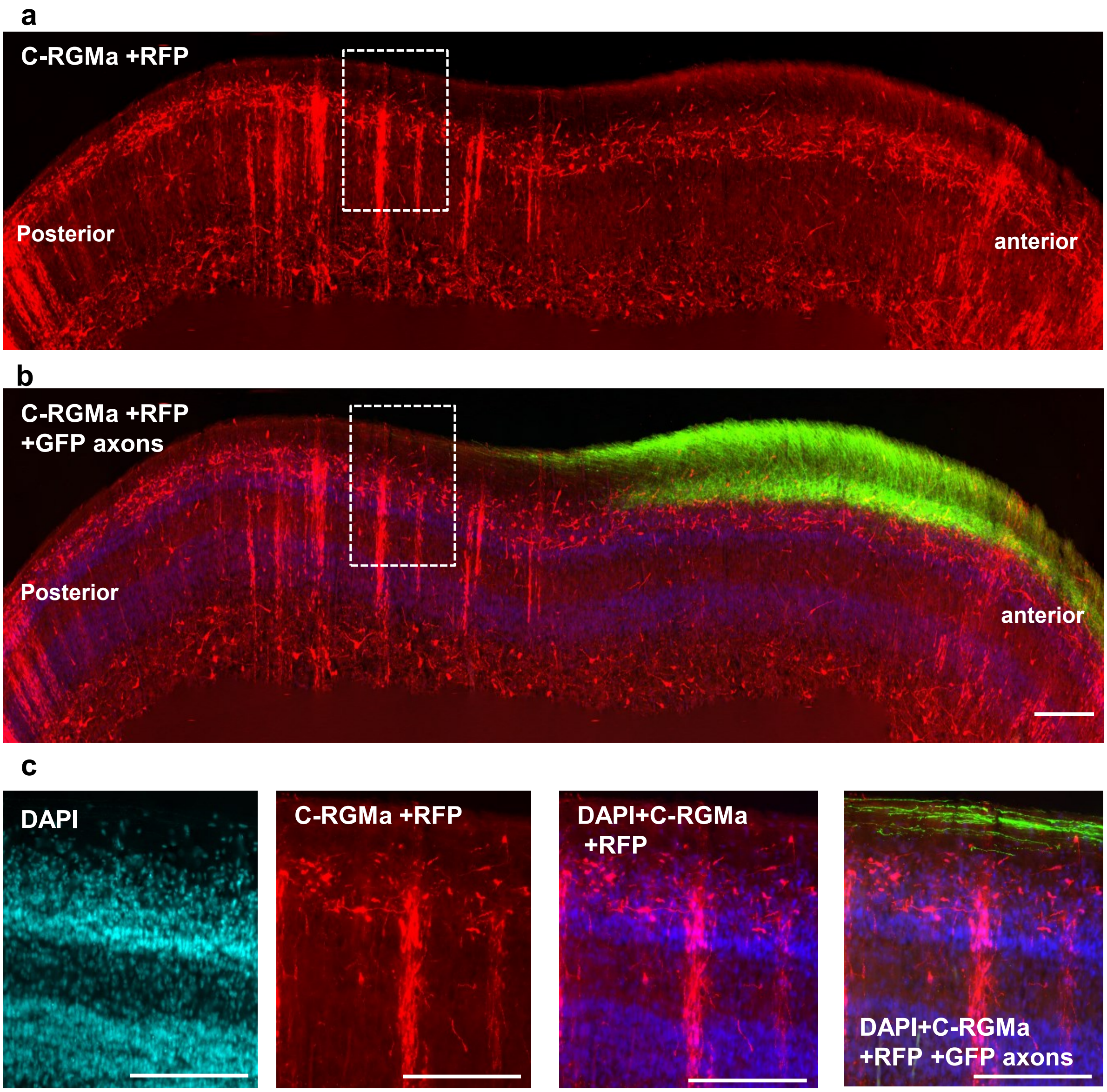

Supp. Figure 11

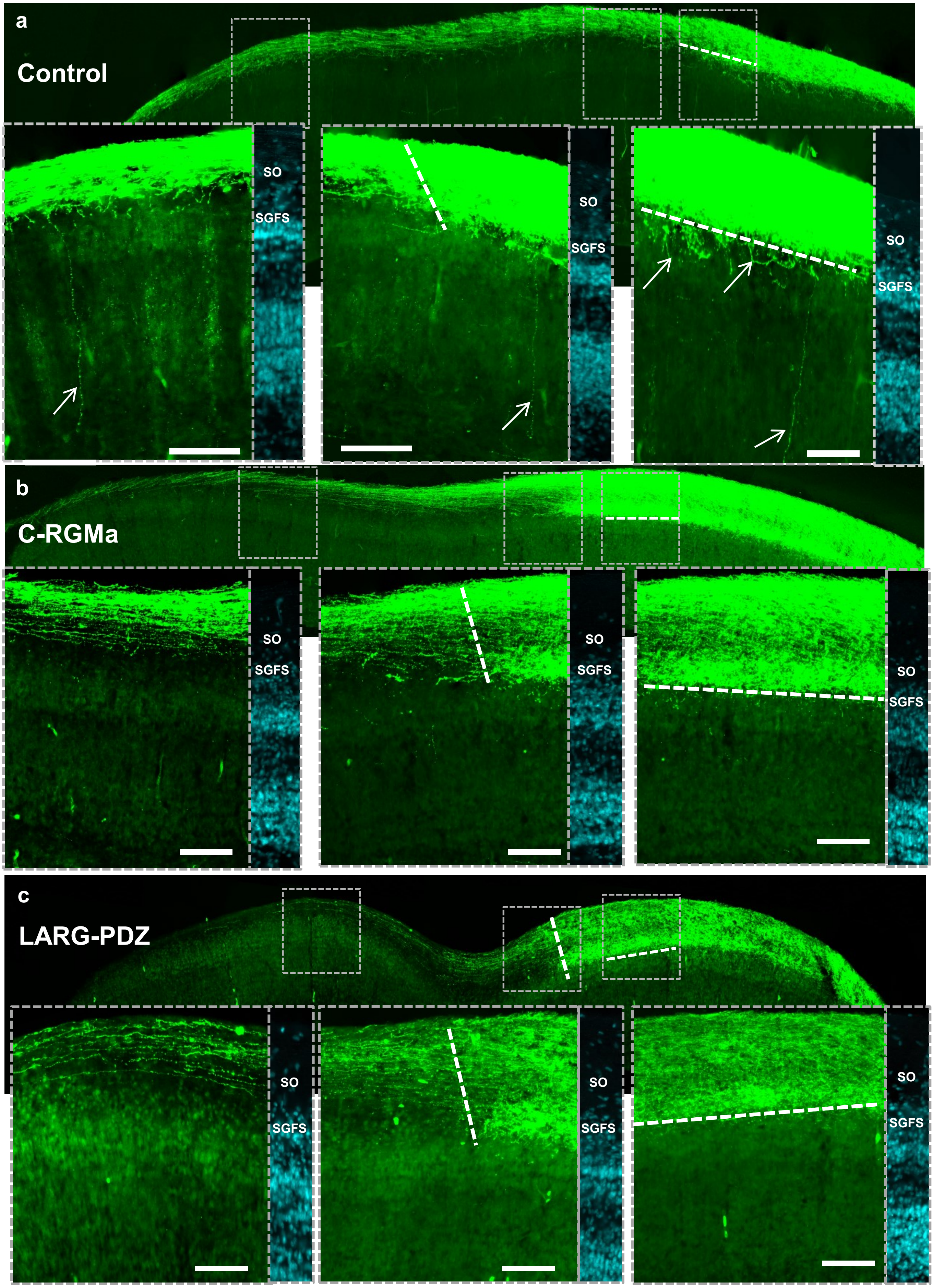

Supp. Figure 12
